# Oral allergy syndrome and risk of food-related anaphylaxis: a cross-sectional survey analysis

**DOI:** 10.1186/1710-1492-7-S2-A2

**Published:** 2011-11-14

**Authors:** Amanda Jagdis, Amin Kanani, Donald Stark

**Affiliations:** 1 Department of Internal Medicine, Faculty of Medicine, University of British Columbia, Vancouver, BC, Canada; 2 Division of Allergy and Immunology, Department of Internal Medicine, Faculty of Medicine, University of British Columbia, Vancouver, BC, Canada; 3 Division of Allergy and Immunology, Clinical associate professor, Department of Internal Medicine, Faculty of Medicine, University of British Columbia, Vancouver, BC, Canada

## Background

Oral Allergy Syndrome (OAS) is an IgE-mediated allergic response to fresh fruits, nuts and vegetables caused by cross-reactivity between pollen allergens and structurally similar food proteins. Alder pollen is a prominent allergen in coastal British Columbia, present at high levels from February- April. We hypothesized that this exposure may lead to increased prevalence of Alder pollen allergy and OAS. We sought to determine our population-based prevalence, cross-reactivity patterns, and incidence of food-related anaphylaxis.

## Methods

A chart review of 574 allergic rhinitis patients seen from January 2010 - June 2011 was performed. 274 OAS patients were invited to participate in an online, telephone or in-person survey. Patients completing the survey in the clinic were invited to undergo a panel of skin prick tests.

## Results

63 patients were surveyed, 14 underwent skin testing. Patient characteristics included: median age=37 (range 20-77), 83% female, 36% atopic dermatitis, 24% asthma. OAS prevalence among seasonal allergic rhinitis patients=242/574 (42%). 14/14 patients were skin test positive for Alder and Birch. The most common OAS foods were apple 44/63 (70%), cherry 37/63 (59%), and peach 38/63 (60%). 28 had epinephrine auto-injector devices; 4 had used their device; 6/10 reactions involved foods that had caused OAS including apple, celery, green pepper, tomato, peanut, walnut.

## Conclusions

In our population, the prevalence of OAS was slightly lower than expected at 42%. The most common OAS/pollen allergy was Alder, correlating with the high Alder pollen exposure in coastal British Columbia. OAS may be associated with serious reactions requiring use of epinephrine.

